# Is cardiovascular MRI equally effective as TEE in evaluation of left atrial appendage thrombus in patients with atrial fibrillation undergoing pulmonary vein isolation

**DOI:** 10.1186/1532-429X-13-S1-P246

**Published:** 2011-02-02

**Authors:** Sandeep Anreddy, Sukhraj Balhan, June A Yamrozik, Ronald B Williams, Mark Doyle, Saundra B Grant, Robert WW Biederman, Vikas K Rathi

**Affiliations:** 1 Allegheny General Hospital, Pittsburgh, PA, USA; 2 Bon Secours Heart and Vascular Institute, Richmond, VA, USA

## Introduction

Patients with atrial fibrillation (Afib) routinely undergo a transesophageal echocardiogram (TEE) for evaluation of the left atrial appendage (LAA) to rule out thrombus prior to undergoing cardioversion or pulmonary vein isolation (PVI). Cardiac MRI (CMR) is now increasingly used for evaluation of these patients for defining pulmonary vein anatomy prior to PVI.

We hypothesized that 2D and 3D non-contrast and contrast CMR is as effective as TEE in evaluating for LAA thrombus while providing simultaneous comprehensive non-invasive evaluation of the pulmonary vein anatomy within a single exam.

## Purpose

## Methods

Afib Pts (n=110, male=82) underwent TEE and non-contrast and contrast CMR prior to undergoing an initial PVI procedure. CMR was performed on 1.5T GE scanner and two blinded CMR experts analyzed the images. The CMR images were analyzed under two categories: 1) the 2D non-contrast cine images showing LAA in 2 chamber and orthogonal views 2) 3D atrial contrast source-images acquired during pulmonary vein angiogram. CMR variables evaluated were the presence or absence of LAA thrombus, quality of images and the results were compared with the results of TEE in a blinded fashion.

## Results

In 62% of pts (n=65) the CMR and TEE studies were performed on the same day, in 37 % of patients (n=39) the mean interval between the two studies was 7 days and in the remaining 6 patients the mean interval was 3+1months. All pts (n=110) were analyzed for the evaluation of LAA thrombus (avg time: 35 min). All (100%) of patients were in Afib and in all (100%) the images were of diagnostic quality (good correlation between the two CMR observers with only one grade difference when in disagreement). Thrombus was absent in 108 of 110 pts on TEE and CMR and present on 2 TEE and CMR images (100% concordance). In 9 cases the 2D CMR images were indeterminate where 3D contrast images were most helpful in the final rule out of LAA thrombus.

## Conclusions

CMR offers a comparable and equally specific alternative to TEE for the complete non-invasive evaluation of LAA thrombus in patients with Afib without the obligate need for sedation, radiation or nephrotoxicity. In one single examination a CMR exam can provide LAA anatomy, rule out thrombus and display the pulmonary veins in patients undergoing a PVI procedure.

